# Disadvantaged Status and Health Matters Networks among Low-Income African American Women

**DOI:** 10.3390/socsci6030108

**Published:** 2017-09-09

**Authors:** Erin Pullen, Carrie Oser

**Affiliations:** 1 Indiana University Network Science Institute, Indiana University—Bloomington, 1001 E State Road 45/46 Bypass, Bloomington, IN 47408, USA;; 2 Department of Sociology, University of Kentucky, 1515 Patterson Office Tower, Lexington, KY 40506, USA; cboser0@uky.edu

**Keywords:** health matters networks, egocentric networks, African Americans, substance use, mental health, criminal justice status

## Abstract

A significant gap in current network research relates to understanding the factors that shape the health matters (HM) networks of marginalized, socially disadvantaged populations. This is noteworthy, given that these networks represent a critical resource for mitigating the adverse health effects of both acute and chronic strains associated with marginalized status. Further, research has suggested that the networks of such populations—especially low-income African American women—are unique, and may operate in substantively different ways than those of other groups. Using two waves of data from a sample of low-income African American women, this research identifies the demographic, health status, and health behavior measures at time one that correspond to HM network characteristics at time two, six months later. This study offers preliminary insights on the relationship between key sociodemographic and health status characteristics of low-income African American women and their HM networks, including criminal justice involvement. Findings reveal that though poorer health status and criminal justice involvement correspond to smaller health matters networks, they also correspond to more active and supportive networks.

## 1. Introduction

Though a robust and important body of research has examined the ways social networks shape health and health behaviors, decidedly less work has investigated how health status and behaviors may shape network outcomes. This is noteworthy given the dynamic relationship between social network factors and health. That is, extant research acknowledges that just as networks are an important force driving health and wellbeing, the structure, content, and function of networks are also influenced by health status and behaviors (Pescosolido 1991; Pescosolido et al. 1998a; Smith and Christakis 2008). When considered as outcomes of interest, social networks may be responsive to stressful life events or health problems, such as depression and hospitalization, as well as socioeconomic and other factors (Perry and Pescosolido 2015; Holmes-Eber and Riger 1990). For example, networks may be disrupted by health crises or behaviors, while other events or behaviors may serve to “activate” existing or new network members to intervene or offer resources (Gage 2013; Perry 2006). Alternatively, individuals may strategically draw on new or existing relationships for emotional and material support, or withdraw from relationships for which they cannot meet reciprocal demands (Thoits 2011).

A significant gap in current research relates to understanding the factors that shape the networks of marginalized, socially disadvantaged populations. Because of the critical role social relationships play in shaping innumerable outcomes among such groups, understanding the ways relevant contextual factors shape these relationships and the resources that flow through ties represents a vital research goal. This study offers preliminary insights on the relationship between sociodemographic and health characteristics among low-income African American women and their health matters (HM) networks. This research responds to this gap and to calls in the literature to bring “the truly disadvantaged”—such as populations living at the intersection of multiple marginalized identities—from the periphery of academic research efforts, to the center (Wilson 2012; Choo and Ferree 2010).

## 2. African American Women’s Networks

Though there has long been substantive interest in the networks of marginalized African American women, research in this area has been slow to develop. Carol Stack’s 1974 volume All Our Kin was one of the first major efforts to examine the structure and function of social relationships among low-income African American women (Stack 1974). Stack’s conclusion, which has been supported by later work, is that African American women living in poverty have close, relatively stable networks comprised of extended kin through which financial, emotional, and other resources are exchanged (Stack 1974; Aschenbrenner 1975; Ellison 1990). These extended ties, developed in part due to the prevalence of single female-headed household and widespread resource deprivation, are critical for coping with chronic stressors (Fowler and Hill 2004; Hays and Mindel 1973). These relationships are also characterized by a high degree of reciprocity, wherein women who rely on their close ties for material and immaterial goods and services critical to their everyday lives are, in turn, obligated to contribute available resources (Stack 1974; Sarkisian and Gerstel 2004). While these exchange networks may represent an adaptive strategy for supplementing limited personal resources, obligations associated with embeddedness in such networks may also have adverse effects on African American women, inhibiting their social mobility and further straining individuals’ already overburdened capacity for coping with exigent life circumstances (Domínguez and Watkins 2003; Jackson 2007).

An important factor that shapes low-income African American women’s networks is the disproportionate incarceration rates they experience. Though white women make up nearly half of the prison population, African American women have twice the rate of imprisonment (Carson 2014). Incarceration has important effects on personal networks, serving to disrupt relationships with those on the “outside” (Rose and Clear 2003). Unfortunately, even after incarcerated women are released, having served time in prison can lead to further marginalization (Schnittker and John 2007). The stigma associated with being a female offender, financial instability, and low self-esteem can all contribute to difficulty maintaining established networks and establishing new relationships upon re-entry, and have lasting effects on health (Clear et al. 2001; Schnittker and John 2007). In addition to overrepresentation in the criminal justice system, which can have deleterious impacts on networks, disparities in the health and wellbeing of low-income African American populations represent a potential threat to the integrity of their social relationships (Wildeman and Western 2010).

## 3. Health Disparities and Health Matters Ties

The health status of low-income African American women represents a prominent concern, as extensive research has described the health disparities experienced by those living at the intersection of multiple disadvantaged statuses (Umberson et al. 2014; Williams and Jackson 2005; Mullings 2014). Findings of this literature indicate that marginalized socio-structural location can contribute to adverse health in myriad ways. One pathway this status shapes health is via greater exposure to discriminatory life events. Research has linked discrimination to depressive symptoms, obesity, and the adoption of unhealthy behaviors, like substance use (Borrell et al. 2013; Schulz et al. 2006). Unaddressed, these experiences and behaviors can generate new or exacerbate existing health problems for which African American women are already disproportionately vulnerable. For example, though African Americans tend to smoke less when compared to whites, they are more likely to perish from smoking and tobacco related causes (CDC 2013; Heron 2013). Low-income African American women are also more likely to experience obesity in their lifetime—as both a product of their decreased access to nutritional food and areas for recreation, and exposure to stressful life events that trigger behaviors like overeating (Williams 2003; Jackson and Knight 2006). Obesity has been linked to chronic stressors low-income African American women are exposed to, including disproportionate rates of incarceration, poverty, and discrimination (Lee et al. 2014; Kelly et al. 1997; Jackson and Knight 2006). Both substance use and obesity are major contributing factors to preventable diseases that disproportionately affect African Americans, including diabetes, hypertension, and heart disease (CDC 2013; Heron 2013; Lee et al. 2014). Taken together, these problems are made worse by limited access and delayed use of preventative health care, and increased use of emergency services (Nishka et al. 2010; Chandler 2010).

In addition to these challenges, women involved with the criminal justice system—disproportionately women of color—have been shown to have more serious health problems compared to the general population, including co-occurring mental and physical health problems and substance use disorders (Lee and Wildeman 2013; Langan and Pelissier 2001; Tuchman 2010). Those who are involved with the criminal justice system are disproportionately from low socioeconomic status backgrounds (Smedley et al. 2003) and research indicates that entry into prison may actually correspond to improved access to health promoting resources (Wilper et al. 2009). In this respect, involvement with the criminal justice system can result in access to resources that were previously out of reach, like substance abuse treatment and mental health services. At the same time, the gains from these resources may be offset by the nature of their usage; for example, when substance abuse treatment is a mandatory condition of one’s sentence, it may be perceived as coercive, thereby considerably limiting its long-term impact (Klag et al. 2005).

Given the general characterization of low-income African American women’s networks as comprised of what Peggy Thoits would term “invested supporters”, including kin and other close ties, these networks represent a critical resource for mitigating the adverse health effects of both acute and chronic strains associated with their marginalized status (2011). However, little is known about how health status patterns interactions with network ties among this population. Research suggests that the social networks of African American women may have a unique effect on their health and wellbeing. African American women typically have larger health networks and are more likely to use informal help when making decisions regarding personal problems than white women (Neighbors and Jackson 1984; Chatters et al. 1989). These ties may also be informal sources of health advice among African American women, simultaneously serving as barriers to formal help-seeking (Chandler 2010). Other research indicates that kin embeddedness predicts happiness and greater life satisfaction among African Americans, both of which are important components of good mental health (Ellison 1990).

## 4. Purpose

Despite important early work by eminent scholars like Stack, there remain significant unanswered questions about the networks of this population. Ultimately, though health disparities among African Americans have long been of interest, there remains a great deal that we do not know about the relationship between their networks and health. A significant gap in this research surrounds the ways networks may be influenced by health problems. This is a particularly salient issue for African American women given the long-standing health disparities that persist despite significant gains in other areas.

The current literature indicates several important trends that motivate further research in this area. On one hand, low-income African American women’s networks are unique with strong evidence suggesting they are relatively stable, comprised of extended kin, characterized by reciprocal exchanges of resources, and linked to various dimensions of well-being. They also appear to be a critical resource for coping with the psychological stress and material constraints of living at the intersection of multiple marginalized statuses. On the other hand, low-income African American women are disproportionately vulnerable to a number of health problems and other factors that may play an important role patterning interactions with network ties. Still—though theoretical and empirical work describing the dynamic relationship between networks and health provide a basis for examining personal networks as both drivers of and responsive to health status—the majority of work in this area has focused on networks as predictors of health related outcomes, while markedly few studies have explored network response to health problems in this population.

This study explores the relationship between health and social status, and interactions with health matters network ties among African American women with varying criminal justice system involvement. Using two waves of data from a sample of low-income African American women, this research identifies which demographic, health status, and health behavior measures at time one, correspond to HM network characteristics at time two, six months later. By identifying significant relationships between individual and demographic characteristics and features of individuals’ health matters networks, this study aims to expand what is known about low-income African American women’s networks. This research also considers the networks of individuals involved with the criminal justice system, a socially vulnerable and disenfranchised group that may need strong networks more than others. Further, identifying the individual and broader social correlates of these marginalized women’s networks may yield important clues about the ways these networks operate in the face of disadvantage and hardship, as well as ways individuals might mobilize available resources in response to health and other problems.

## 5. Materials and Methods

### 5.1. Data

Data used in this research are from the Black Women in the Study of Epidemics project (B-WISE), a longitudinal study that collected data from 2008 to 2014 on 643 African American women. The overall goals of the project were to identify disparities in the health and health service utilization of drug using and non-drug using African American women across criminal justice status. A total of 643 African American women were recruited for the study and completed an intake interview, including 240 prisoners, 197 probationers, and 206 community-based participants (not under criminal justice supervision when recruited). A stratified sampling technique was used such that approximately half of each sample were drug users, while the other half were non-drug users. This sampling strategy was selected due to the high rates of drug use among women who were incarcerated or under probationary supervision. After completing the first interview at Wave 1, participants completed follow-up interviews at 6, 12, and 18 months after intake or, for those recruited while incarcerated, after their release. These interviews are Wave 2, Wave 3, and Wave 4, respectively. Follow-up rates for eligible participants were excellent across all three follow-up interviews (Wave 2 = 94%; Wave 3 = 92%; Wave 4 = 90%).

Data for this research are taken from two time points collected 6 months apart, representing Wave 3 and Wave 4. These time points were selected because network measures were captured only during the final wave of data collection. For the present study, Wave 3 data is referred to as “baseline” and Wave 4 is referred to as “follow-up”. The health matters network inventory was not included until midway through the study, therefore ego network data are only available for 344 participants.

African American women were recruited from three Kentucky prisons and seven probation offices. Community-based respondents were recruited through flyers and advertisements in local newspapers. Study flyers were posted in areas with the highest percentage of African American residents based on Census data. Eligibility was limited to women who self-identified as African American, were at least 18 years old, and were willing to participate. Women recruited as part of the prison sample were also required to be incarcerated at the time of the Wave 1 interview and eligible for release within 60 days of this interview. With the support of the Kentucky Department of Corrections, study staff were provided with a list of the female prisoners, and women meeting eligibility requirements were invited via letter to attend a study informational session, where interested parties were screened and invited to participate. To recruit participants for the probation sample, trained interviewers approached all African American women entering probation offices during report days. These women were screened, with interested and eligible women scheduled for a Wave 1 interview. Interviews with women recruited from probation offices and the community were conducted in private rooms of public venues, such as university research offices, libraries, or community based organizations. All participants were screened prior to enrollment in the study to determine their drug use status in an effort to ensure approximately half of all samples were non-drug users. All Wave 1 interviews were conducted face-to-face, lasted about two hours, and were completed by trained African American female interviewers. Follow-up interviews were completed primarily face-to-face, but in some instances, via telephone to accommodate participants. All protocols were approved by the Institutional Review Board and a federal Certificate of Confidentiality was obtained. No data was shared with the Department of Corrections.

### 5.2. Measures

#### 5.2.1. Dependent Network Variables

The health matters network name generator has emerged as a useful tool for understanding the relationship between ego networks and health. Research utilizing the HM network name generator has found that it is a more accurate representation of the networks that individuals confer with regarding health (compared to the important matters network) and that these networks can serve important functions as individuals evaluate their health status, make help-seeking decisions, and recover from threats to their health and wellbeing (Perry and Pescosolido 2010, 2015). This research utilizes this tool, and the outcome measures used in these analyses are taken from the HM network generator, described in greater detail below.

##### Health matters network size.

A HM name generator was administered at follow-up, eliciting a list of individuals respondents talk to about health (Pescosolido and Perry 2010). Specifically, respondents listed the name of ties they “talk to about health problems when they come up” and “can really count on” when they have physical or mental health problems and ties that always talked to them about their health and try to get them to change their behaviors or see a health professional (Perry and Pescosolido 2010). Taken together, ties that were named as fulfilling these functions were considered members of participants’ health matters networks. HM network size ranged from 0 (about 22% of participants) to 5 (less than 1%), with a mean HM network size of 1.39 (SD = 1.09).

##### Discussion frequency.

Respondents also reported how often they discussed health matters with each network member. Likert responses ranged from 1 (rarely/neve r) to 6 (almost every day). For each participant, the mean discussion frequency for all network members was taken and this value was standardized before regression. This measure therefore represents the mean discussion frequency with all HM members at follow-up, ranging from 1 to 6 (x ® = 4.10; SD = 1.36). Because some study participants reported having no one to talk to about their health, they were not included in analyses examining this outcome, yielding a smaller sample size (N = 265).

##### Encouragement to improve health.

In addition to network structure and strength of relationships, it is also important to capture the type of information that “flows” through these networks so that it is possible to consider what influence they might have on behavior. Therefore, at follow-up participants were asked how often HM ties encouraged them to “see a health professional, to stop doing things that are bad for [their] health, or to begin healthy behaviors”. For each participant, the mean level of encouragement from all HM network members was calculated and this value was standardized before regression. The mean HM network encouragement ranged from 1 to 6 (x ® = 4.23; SD = 1.59). Participants who reporting having no one talk to about health matters were excluded, yielding a smaller sample size (N = 265).

##### Health discussants.

Participants were also asked about the presence of health discussants in their networks. Specifically, for each person listed, respondents were asked if they were someone whom they “talk about health problems when they come up” and “really count on when [they] have physical or emotional problems”. The number of health discussants was then dichotomized such that any respondent with 1 or more health discussants was coded 1 (77%), while those without health discussants were coded 0 (23%).

#### 5.2.2. Independent Variables

##### Socio-demographic variables.

Age at baseline is coded in tens of years. Participant age ranged from 18 to 68 years (x ® = 35; SD = 1.12). Marital status at baseline is a dichotomous variable, coded 1 for legally married or living as married (14%) and coded 0 for all other categories (86%). Baseline household income is coded to the midpoint, in tens of thousands of dollars. Participants reported their income as a range (e.g., $5000 to $9999), which was then coded to the midpoint (e.g., $7500), and converted to tens of thousands of dollars (e.g., 7.5). Income ranged from 2.5 (i.e., $0 to $4999) to 87.5 (i.e., $75,000 or more), with a mean score of 10.65 (SD = 13.65). Education at baseline is coded in years and ranges from 5 to 20 (x ® = 11.92 years (SD = 2.18)).

##### Criminal justice status.

Given the sampling strategy for this data and the disproportionate number of health problems among those in the criminal justice system, two control measures for criminal justice status are used. Two dichotomous variables, *prison* and *probation,* are coded 1 for being recruited while incarcerated or on probation respectively, with recruitment while not under criminal justice supervision serving as the reference category.

##### Substance Use.

Additionally, because of the overrepresentation of drug users and the aforementioned link between substance use and adverse health outcomes, three measures of substance use at baseline are included in these analyses. Alcohol use is measured by a dichotomous variable coded 1 for participants who report using alcohol to intoxication one or more times during the past six months (80%), and 0 for women who did not. Use of crack or cocaine is measured with a dichotomous variable coded 1 for participants who reported using either crack or cocaine in the past six months (18%), and 0 for women who did not. Crack and cocaine use are selected for these analyses because they have disproportionately affected African Americans in the United States, and contribute to disrupted social relationships and relationship conflict (Golub et al. 2010; Hendricks and Wilson 2013). Finally, participants who used tobacco daily are given a value 1 (57%), while those who do not are coded 0.

##### Physical Health.

Two broad measures of physical health status are used. These measures both relate to body mass, as obesity has been linked to chronic stressors that low-income African American women are disproportionately exposed. Body mass is calculated using self-reported height and weight at baseline with the standard Body Mass Index (BMI) formula: BMI = (weight*703)/height^2^. BMI scores range from 15.8 to 57.4 (x ® = 32; SD = 8.02). Given the limitations of relying solely on BMI score to assess general physical health, an additional measure of physical activity is also included. Exercise is coded as days per week the respondent reported getting at least 30 minutes of physical activity on average over the 6 months prior to baseline. Scores range from 0 (20%) to 7 (29%) (x ® = 3.7; SD = 2.65).

##### Other Health Status.

Two additional health status measures are used. Mental health is examined using a self-reported measure of depression at baseline. This measure is coded 1 if participants reported experiencing two or more weeks of serious depression in the past 6 months (21%) and 0 if they did not (79%). Additional measures of mental health status were also initially examined, but were ultimately excluded due to multicollinearity. A final health status measure for high acuity healthcare encounters is considered with a measure of overnight hospital stays reported for the past 6 months at baseline. This count measure ranges from 0 (91%) to 10 (< 1%).

## 6. Analyses

First, summary statistics are presented for the total sample, and each level of criminal justice supervision in Table 1. Next, differences on the baseline independent variables between participants who reported 0 health matters ties and 1 or more health matters ties are explored. Test statistics used to determine significant differences include the Wilcoxon-Mann-Whitney test for non-normally distributed interval or ordinal variables, t-tests form normally distributed interval or ordinal variables, and chi-square tests and Fisher’s exact test for categorical variables.

To examine which independent measures at baseline (Wave 3 of the B-WISE Study) are significant predictors of social network outcomes at follow-up (Wave 4 of the B-WISE Study), regression analyses are performed. Specifically, Poisson regression is used to predict the size of health matters networks at follow-up, linear regression is used to predict both discussion frequency and encouragement to utilize services, and logistic regression is used to predict the dichotomous health discussant outcome. To visually represent findings regarding the role of criminal justice status as a predictor of health matters ties mean encouragement to improve health, predicted probabilities are shown.

## 7. Results

### 7.1. Summary Statistics

Table 1 provides the summary statistics for both the independent and dependent variables examined in this study, presenting an average for the total sample (N = 344) and averages for participants recruited for the community, probation, and prison samples. Generally speaking, community based women have larger networks (1.68) at follow-up compared to women recruited while in prison (1.18) or on probation (1.43). Participants recruited from the community, however, have less frequent discussion with their HM ties (3.87) and receive less encouragement to improve their health (3.65), on average, compared to women from the prison (4.04 and 4.55, respectively) and probation samples (4.36 and 4.34, respectively).

As Table 1 also shows, there are differences across demographic and substance use measures for participants based on their criminal justice status at the time of recruitment. Though participants are relatively similar in age and education across the three samples, only about 9% of women recruited while incarcerated are married or living as married, compared to 15% of probationers and 21% of community based participants. Annual mean household income also differs across participants, with participants recruited while under no criminal justice supervision having a higher annual income ($15,000), on average, compared to women on probation ($10,600) or in prison ($7960) at the time of study recruitment. Though community, probation, and prison samples have similar incidence of crack or cocaine use, women from the community sample are more likely, on average, to report using alcohol to intoxication (29%) compared to those from the probation (13%) or prison samples (14%). Conversely, they have lower rates of daily tobacco use (38%) compared to women incarcerated (63%) or on probation (65%) at the time of recruitment.

Measure of health status and their averages across recruitment sample are also presented. While the three samples have similar average BMI scores, women recruited from the community exercise slightly more frequently on average (about 4 days per week), than participants from the prison and probation samples (3.56 and 3.62, respectively). Additionally, about 23% of women recruited while in prison report experiencing a period of depression that lasted 2 weeks or longer in the past 6 months, compared to 22% of probationers and 17% of women from the community. Finally, about 10% of women recruited while incarcerated report having spent a night in the hospital in the past 6 months, while about 8% of women from the community and probation sample report an overnight hospitalization.

### 7.2. Bivariate Statistics

Table 2 displays descriptive statistics of the independent variables for participants who reported no health matters ties, and those who report at least 1 health matters tie. As this table indicates, for the majority of the independent variables used, there are not significant differences between women who reported no health matters ties and those that reported 1 or more ties. However, as might be expected, women with 1 or more HM ties are significantly more likely to be married or living as married, compared to those with no health matters ties (16% compared to 8%). Women without HM ties are also significantly older than those with one or more ties (38 years on average, compared to 35 years). Women without HM ties report significantly lower rates of alcohol use to intoxication (69%), compared to those with 1 or more HM ties (84%). Finally, participants without HM ties are significantly more likely to report 2 or more weeks of depression at baseline than women with HM ties (29% compared to 19%), and significantly more likely to report having an overnight hospital stay than women with 1 or more HM ties (16% compared to 7%).

### 7.3. Network Size

Table 3 shows the results of Poisson regression models predicting the size of health matters networks at follow-up with baseline demographic and health status measures (incidence rate ratio [IRR] and standard error presented). Across all models, women who were married or living as married in the previous wave are predicted to have larger health matters networks at follow-up compared to women who were single, divorced, separated, or widowed. As Model 1 demonstrates, women who were recruited while incarcerated have significantly smaller health matters networks at follow-up than women recruited while under no criminal justice supervision (IRR = 0.72, *p* < 0.01). Model 2 finds that women reporting alcohol use to intoxication at baseline have larger HM networks at follow-up (IRR = 1.33, *p* < 0.05), while those who report using crack or cocaine have smaller HM networks at follow-up (IRR = 0.76, *p* < 0.05). According to Models 3 and 4, neither measures of BMI and physical activity nor other health status measures at baseline significantly predict health matters network size at follow-up. Finally, the findings of the full model—Model 5—demonstrate the significant effects of marital status, recruitment while incarcerated, and use of crack or cocaine over and above the other health status measures considered (IRR = 1.40, *p* < 0.01; IRR = 0.77, *p* < 0.05; and IRR = 0.75, *p* < 0.05, respectively).

### 7.4. Discussion Frequency

The findings presented in Table 4 show the significant baseline predictors of mean discussion frequency with health matters network members at follow-up. Across all the restricted models, income is a significant predictor of mean discussion frequency with HM ties such that each unit increase in household income at Wave 3 predicts a 0.01 standard deviation decrease in mean discussion frequency with health matters network members at Wave 4 (*p* < 0.05). Model 1 also indicates that being on probation when recruited predicts a 0.33 standard deviation increase in mean frequency of discussion with HM ties, compared to women recruited while under no criminal justice supervision. According to Model 3, each unit increase in BMI score at baseline predicts a 0.02 standard deviation increase in the mean frequency of discussion with HM network members at follow-up (*p* < 0.01). Similarly, women who experienced two or more weeks of depression or who have had an overnight hospital stay in the previous wave have greater predicted mean discussion frequency with HM network members compared to women who did not experience depression or have an overnight hospital stay, net the controls (β = 0.40, *p* < 0.05 and β = 0.51, *p* < 0.05, respectively).

Model 5 shows the significance of these measures considered together. Though income is significant in the restricted model, it does not achieve significance in the full model. However, all other significant relationships from the restricted models hold.

### 7.5. Encouragement to Improve Health

Table 5 presents the results of regression models predicting mean encouragement to see a health professional, change negative health behaviors, or begin healthy behaviors at follow-up from HM network members. Coefficients and standard errors are shown. Across all models, as household income at baseline increases, women receive significantly less mean encouragement from HM network members to improve their health. According to Model 1, women who are recruited while under criminal justice supervision receive greater encouragement from HM network members to improve their health, compared to women who were recruited from a community sample (β = 0.48, *p* < 0.01 and β = 0.39, *p* < 0.05 for prison and probation recruitment, respectively). Model 2 considers the influence of three types of substance use on level of encouragement to improve health from HM network members, finding that these measures do not appear to significantly influence encouragement received from HM ties at follow-up. Model 3 indicates that as BMI score at the previous wave increases, participants are significantly more likely to receive encouragement to improve their health at follow-up (β = 0.01, *p* < 0.05). In Model 4, net the demographic control measures, women who experienced two weeks or more of depression in the previous wave receive more encouragement from HM network ties at follow-up than women who do not report depression (β = 0.40, p < 0.01). Results of Model 4 also indicate that women who report an overnight hospital stay at the previous wave receive greater encouragement to improve their health at follow-up than women who did not have a hospital stay (β = 0.46, *p* < 0.05).

**Figure 1.**
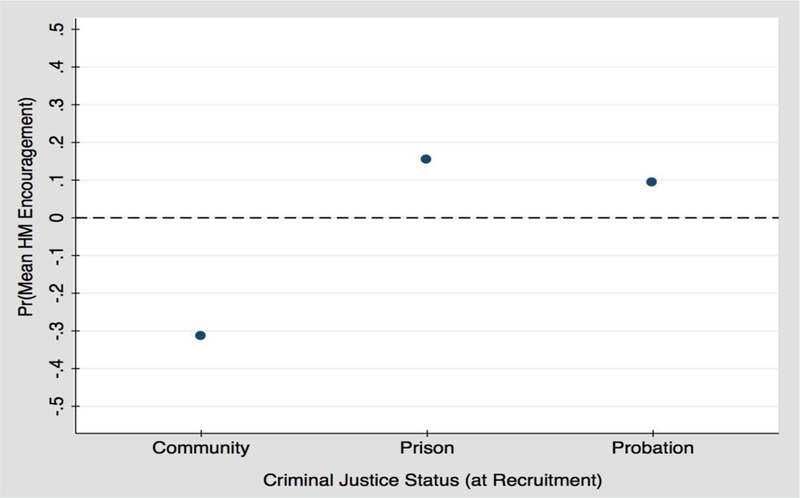
Predicted probability of encouragement from HM ties by criminal justice status.

The results of the full model, Model 5, indicate that women recruited while under criminal justice supervision, experienced two or more weeks of depression, or were hospitalized overnight in the previous wave, received significantly greater mean encouragement to improve health. As a visual companion to these results, Figure 1 shows the predicted values of mean encouragement from health matters ties by criminal justice status. As this figure demonstrates, women involved with the criminal justice system have greater predicted encouragement from HM ties to adopt healthy behaviors at follow-up, compared to community-based participants.

### 7.6. Health Discussants

Table 6 indicates the significant predictors of having a health discussant in participants’ HM network. As shown in Model 1 and across all models, as age at baseline increases, the predicted odds of having a health discussant in one’s network decrease. Also observed across all models, compared to women that are single, divorced, or widowed, those who are married or living as married in the previous wave are predicted to be more likely to have a health discussant in their network at follow-up. Model 1 also indicates that women who are recruited while on probation have lower predicted odds of having health discussants in their network when compared to community-based women (OR = 0.37, *p* < 0.05).

Models 2 and 3 demonstrate that neither substance use nor BMI and physical activity significantly predict having a health discussant in one’s network. However, as shown in Model 4, women who had an overnight hospital stay in the previous wave were significantly less likely to have a health discussant at follow-up. In Model 5, all significant predictors identified in the restricted models hold.

## 8. Discussion

The purpose of this research was to examine the relationship between select health status and behavior measures and health matters network characteristics among low-income African American women. These analyses reveal that interactions with health matters network ties significantly correspond to health behaviors and measures of health status among this population. Specifically, as individuals manage their health and adverse life circumstances, they have more discussions with and receive more encouragement from those in their health matters networks. Evidence from this research suggests that though the health matters networks of individuals with poorer health may be smaller in some instances, they may also be more active in terms of discussion frequency and encouragement to adopt healthy behaviors. Taken together, the results of this research highlight a number of health status and behavior factors corresponding to low-income African American women’s involvement with their health matters network ties. Importantly, this work responds to gaps in the existing literature regarding the factors that influence ego network structure and function among this disadvantaged and underserved group.

### 8.1. Health Status and Health Matters Networks

First, the results of this research demonstrate that health status and health behaviors at baseline have a significant relationship to individuals’ health matters networks at follow-up. For example, those with higher BMI scores at baseline tended to have more frequent discussions with health matters ties at follow-up. Similarly, those with higher BMI at baseline, on average, received greater encouragement to improve their health from health matters ties at follow-up, though this relationship is only significant in the restricted model. These results suggest that being overweight may prompt individuals to interact with their health matters network ties, possibly turning to them with greater frequency to talk about health. In turn, these ties may be more likely to provide support and encouragement to change unhealthy behaviors. This finding is of special relevance to African American women, as obesity represents a major public health problem among this group. Compared to white women, African American women are 80% more likely to be obese (Levi et al. 2011). Obesity also exacerbates other health problems that African Americans are disproportionately likely to experience, including diabetes and hypertension (CDC 2013). This research suggests that rather than being normalized among African American women, BMI may serve to motivate discussions about health, and prompt encouragement to adopt more healthy behaviors. It is important to note that though health matters ties *may* be activated by individuals in response to perceived concerns related to their BMI score, it is unclear if the involvement of these ties is actually predictive of improved health status.

Like BMI score, the other health indicators examined here significantly correspond to greater involvement with HM network ties. That is, reporting two or more weeks of depression or being hospitalized overnight at baseline predict more frequent discussion with HM ties at follow-up, as well as health promoting encouragement at follow-up. Both predictors represent health needs and these findings suggest that network ties may be a critical resource individuals draw on when managing these problems. These results suggest that among low-income African American women, experiencing depression and hospitalization may prompt discussion with HM ties, and that this discussion may also include suggestions from these ties to improve their health. The results of bivariate statistics conducted – indicating that participants without HM ties also had significantly greater rates of depression and high acuity health care encounters than those with ties – also suggest that alters may serve as an essential resource mitigating the severity of health problems.

The findings presented here regarding depression are particularly important. Existing research argues that expressing vulnerability, even in times of hardship, may be perceived as weakness, while projecting an image of strength is the preferred cultural norm among African American women (Woods-Giscombe 2010; Poussaint and Alexander 2000; Boyd 1998). Though this may be the case in some circumstances, this research shows that rather than concealing periods of depression and health vulnerability, the low-income African American women sampled conferred with their HM networks and were provided with support to pursue health-promoting activities. This suggests that, though other relationships may be jeopardized or threatened by confiding such vulnerabilities, HM ties may be an important outlet for this type of information.

### 8.2. Criminal Justice Involvement and Networks

Because the data used in these analyses includes African American women with varied levels of criminal justice involvement, this permitted examination of how such involvement relates to interactions with HM networks. This is of particular relevance because, as past research has shown, women involved with the criminal justice system have a higher prevalence of mental and physical health problems compared to women who have no involvement (Moloney et al. 2009). Further, detention can also have deleterious effects on the mental and physical health of African American women (Freudenberg 2002; Rose and Clear 2003). Though incarceration, for some, may serve as a gateway to accessing basic health care—especially substance abuse treatment—it can also serve to disrupt personal health and support networks in ways that may have significant impacts on health and wellbeing (Hochstetler et al. 2010).

The results of this research support and extend these findings. Results indicate that women who are incarcerated tend to have smaller networks at follow-up than women without criminal justice involvement, while those on probation at the time of recruitment are significantly less likely to have a health discussant in their HM network at follow-up. Though these results align with past research and imply possible network disruption, results also suggest that the HM networks of African American women may be more engaged given such involvement. That is, findings show that women involved with the criminal justice system engage in more frequent discussion with HM ties, and, importantly, are more likely to report receiving encouragement to see a health professional, change negative health behaviors, or begin healthy behaviors from these ties. This suggests that though fewer HM ties remain involved in the networks of women with criminal justice involvement, these alters are more invested in shaping their health. Most significantly, these ties are a source of health-promoting encouragement—a particularly important resource, as populations reentering the community after incarceration are vulnerable to a number of negative health behaviors, including substance use and depression.

Though the increased involvement of health matters network ties may be, in part, a response to the poorer health these women experience generally, the significance of criminal justice involvement in predicting encouragement from health matters alters remains even when additional measures of health status are included as covariates. That criminal justice involvement corresponds to health-promoting encouragement from HM ties is a surprising finding. However, it may be that the ties remaining involved with women who were incarcerated or on probation, despite numerous barriers (e.g. confinement or restricted mobility) or threats to their relationship (e.g. reduced social desirability or inability to meet reciprocal network demands), are more resilient and invested in the long-term well-being of these women than more casual ties. Further, involvement in the criminal justice system may highlight certain health problems, like substance abuse and mental health concerns, which could prompt interactions with HM networks. Those involved with the criminal justice system may also benefit from positive relationships with case managers, probation officers, social workers, or others that become part of their network as they re-enter the community. Alters of this kind may monitor the health and wellbeing of their clients and engage in discussions about their health, suggesting actions to promote positive changes, and even spurring conversations with other network ties about these issues. In any case, further research is warranted as the involvement of such ties has the potential to enhance intervention and other efforts that address health disparities among this vulnerable population as they re-enter communities.

### 8.3. Socioeconomic Status and Networks

Finally, this research also sheds light on the role of key sociodemographic characteristics in shaping interactions with HM ties. As might be expected, married/cohabitating women have larger networks and they are also more likely to report having a health discussant in their HM network, as compared to their non-married counterparts. These findings suggest that spouses or serious romantic partners serve as ties that African American women can “really count on” when managing their health, though they may or may not offer health promoting encouragement. Though this research shows that spouses may be a resource drawn on by low-income African American women as they manage their health, it is important to note that the rates of marriage among African Americans are low both within the data examined here and at a national level (Dixon 2009; Banks 2011). Due to a number of possible reasons, including low-rates of interracial partnership and marriage, an imbalanced ratio of available, socially desirable African American men to women, and mass incarceration, African Americans are among the least likely to get and stay married (Dixon 2009; Banks 2011). In all, though romantic partners may be valuable resources for *some* low-income African American women, it is important not to overstate their significance for African American women more generally.

In addition to marital status, household income and age emerged as significant correlates of health matters network characteristics. Income is significantly related to both encouragement to engage in health-promoting activities and discussion frequency with HM ties. Specifically, higher income corresponds to less frequent discussion with, and less health-promoting encouragement from, HM alters. It may be that more affluent participants have fewer health problems that warrant discussion or encouragement to change, thereby partially explaining this negative finding. This interpretation aligns with extant research that finds as income increases, so too do the availability and use of preventative and other health services, thereby driving lower rates of unaddressed health needs among more affluent women (Dubay and Lebrun 2012).

However, it is worth noting that though research indicates income and life expectancy are positively correlated among African American women, it also suggests that greater socioeconomic status for African Americans may also mean greater exposure to discrimination and hostility as African Americans navigate “white spaces”—increasing stress and driving related maladies (Anderson 2014). Because this research relies on a sample of predominantly low-income African American women, a more nuanced view of the complex interplay between income, social status, health, and social networks is beyond our scope. The health and wellbeing of middle and higher income African Americans and the specific stressors that are associated with their status, potentially shaping both their health and networks, are an important area for future research.

This research also indicates that as age increases the likelihood of having a health discussant in one’s HM network significantly decreases. This relationship holds across all models and represents a troubling finding, as older African American women have significant health needs. That older women are significantly less likely to have ties that they can rely on when health problems arise suggests they may have to navigate health problems and make decisions without conferring with others. This may, in turn, increase their vulnerability to adverse outcomes as they manage their health.

### 8.4. Limitations and Future Research

There are important study limitations that should be acknowledged. Though all samples were recruited in the state of Kentucky, the communities that incarcerated participants returned to upon release may not exactly mirror those from which the probation and community sample were drawn. Investigators did try to minimize this by focusing community and probation recruitment efforts on areas with the highest proportion of African American residents in the state. Additionally, the B-WISE data are not representative of African American women nationally. The data are, however, a relatively balanced representation of low-income urban African American women across criminal justice status. The stratified sampling technique, with three samples (community, prison, and probation) and approximately half of all participants reporting drug use at the baseline interview, presents a unique opportunity to examine how marginalized statuses and criminal justice involvement shape health matters networks. Because low-income African American women are an understudied and underserved population, conducting research of this kind—especially over more than one wave of data collection—represents an important contribution. Another limitation to this research is the duration of the study. Because only two waves of data, collected approximately 6 months apart, were used for these analyses, these findings do not necessarily reflect the factors that predict health matters network characteristics over longer periods of time.

Finally, an important limitation of this study is the availability of only a single wave of health matters network data. For this reason, these findings must be interpreted carefully. Rather than arguing the results presented here represent causal pathways, they should be accepted as a *preliminary* indication of such relationships. Furthermore, though health concerns may pattern more interaction with network ties, this research does not provide insight on how this interaction may or may not influence health status and health behaviors. It is possible, for example, that “encouragement” from network ties may actually be perceived negatively as coercion or hassling, as has been found in previous research (Pescosolido et al. 1998b).

Future research should address these limitations by examining data that is nationally representative or representative of women of color in difference contexts, for whom patterns of health network activation and disruption may differ. Additionally, further research should examine these patterns over longer periods of time, preferably using multilevel or structural equation modeling techniques. Finally, though this study begins to address the lack of targeted research regarding the role of ego networks in shaping health outcomes, future studies should continue to examine how various resources “flow” through networks and the mechanisms underlying the dynamic relationship between health and personal networks. Pursuing the collection of egocentric network data on special populations, especially longitudinal data that allows researchers to examine network dynamics and change, is a vital step in furthering this essential research agenda.

## 9. Conclusions

In all, these findings add to the growing body of research examining the relationship between health and networks, and provide new insights on the networks of low-income African American women. The findings of this research align with past findings that identify and describe the phenomenon of health matters network activation in response to health problems (Perry and Pescosolido 2015), even though they represent only a brief time frame. Though research has long suggested that the relationship between health and ego networks is dynamic, with network structure and function both shaping and being shaped by health, the research examining the significant association between disruptive life events and network interactions has been slow to develop. This is especially true for the population examined in this study. This research begins to clarify how a number of disruptive life events – including incarceration, substance use, and depressive symptoms – may prompt the involvement of HM networks. Because the relationship between adverse life events like criminal justice involvement and poor health can be and often are related, it may be difficult to determine whether interaction with network ties is a response to a disruptive event or that event’s health consequences. These analyses provide initial support for the relationship between these measures, though additional longitudinal research is needed to determine the causal direction. Further, these results demonstrate how health status and sociodemographic context pattern relationships with HM ties among low-income African American women, a group whose networks are unique and inadequately understood and who demonstrate both significant health needs and disproportionate exposure to adverse life events (Neighbors and Jackson 1984; Ellison 1990; Chatters et al. 1989).

The results of this research reveal that though poorer health status and greater health need may predict smaller networks, they also predict more active, engaged networks. Specifically, though criminal justice status corresponds to fewer networks ties, these results suggest that the remaining ties may be more likely to intervene or be actively involved. Health matters networks are also more likely to be involved when considering more global measures of health status, such as BMI, resulting in those with higher BMIs having more frequent contact with and greater encouragement from health matters networks to improve their health. Overall, our findings provide preliminary evidence of the responsive nature of low-income African American women’s ego networks in the face of crisis and health problems. It appears that when disruptive conditions and events emerge, a few network members become active supporters of decision-making and providers of information and advice to promote healthier behavior.

## Figures and Tables

**Table 1.** Demographics for the total sample and the community, probation, and prison samples

	Total Sample		Community Sample		Probation Sample		Prison Sample	
	
	N	Mean/Prop	N	Mean/Prop	N	Mean/Prop	N	Mean/Prop
Outcome Measures (Follow-Up)								
HM ^a^ Network Size	344	1.39 (1.10)	86	1.68 (1.05)	121	1.43 (1.20)	137	1.18 (0.98)
HM Discussion Freq (mean)	268	4.10 (1.36)	73	3.87 (1.22)	89	4.36 (1.47)	106	4.04 (1.34)
HM Encouragement (mean)	268	4.23 (1.59)	73	3.65 (1.55)	89	4.34 (1.64)	106	4.55 (1.47)
HM Discussant in Network	344	77.33 % (0.42)	86	84.88 % (0.36)	121	71.90 % (0.45)	137	77.37 % (0.42)
Demographics (Baseline)								
Age (years)	344	35.45 (1.12)	86	36.63 (1.49)	121	34.40 (1.01)	137	35.99 (0.94)
Married/Living as Married ^2^	344	14.24 % (0.35)	86	20.93 % (0.41)	121	14.88 % (0.36)	137	9.49 % (0.29)
Income (tens of thousands)	341	10.65 (13.65)	85	15.00 (18.46)	121	10.60 (13.27)	135	7.96 (9.07)
Education (years)	344	11.92 (2.18)	86	12.99 (2.17)	121	11.63 (2.03)	137	11.50 (2.12)
Substance Use (Baseline)								
Alcohol Use to Intoxication	344	17.44 % (0.38)	86	29.07 % (0.46)	121	13.22 % (0.34)	137	13.87 % (0.35)
Crack/Cocaine Use	342	17.54 % (0.38)	86	18.60 % (0.39)	120	15.83 % (0.39)	136	18.38 % (0.39)
Daily Tobacco Use	342	57.60 % (0.49)	86	38.37 % (0.49)	120	65.00 % (0.48)	136	63.24 % (0.48)
Physical Health (Baseline)								
BMI ^b^	343	32.00 (8.02)	86	31.94 (8.00)	120	32.25 (8.24)	137	31.83 (7.90)
Exercise (days/week)	343	3.81 (2.65)	86	4.06 (2.39)	120	3.62 (2.78)	137	3.56 (2.70)
Other Health Status (Baseline)								
Depression (2 weeks +)	343	21.28 % (0.41)	86	17.44 % (0.38)	120	21.67 % (0.41)	137	23.36 % (0.42)
Overnight Hospital Stay	343	9.04 % (0.29)	86	8.24 % (0.28)	120	8.33 % (0.28)	137	10.22 % (0.30)
Total Observations	344		86		121		137	

a Health Matters;

b Body Mass Index.

**Table 2.** Differences in independent predictors for participants reporting no health matters network ties to those with one or more ties

	No Health Matters Ties		1+ Health Matters Ties		Test Statistic ^1^
	
	N	Mean/Prop.	N	Mean/Prop.	
Demographics					
Age (tens of years)	75	38.32	269	34.65	**
Married/Living as Married ^2^	6	8.00 %	43	15.99 %	*
Income (tens of thousands)	75	10.77	266	10.62	
Education (years)	75	12.09	269	11.87	
Criminal Justice Status					
Prison	30	40.00 %	107	39.78 %	
Probation	32	42.67 %	89	33.09 %	
Substance Use					
Alcohol Use to Intoxication	52	69.33 %	223	83.90%	**
Crack/Cocaine Use	18	24.00 %	45	16.73 %	
Daily Tobacco Use	47	62.67 %	150	56.18 %	
Physical Health					
BMI	75	31.82	268	32.06	
Exercise (days/week)	75	4.04	268	3.61	
Other Health Status					
Depression (2 weeks +)	22	29.33 %	51	19.03 %	*
Overnight Hospital Stay	12	16.00 %	19	7.09 %	*
Total Observations	75	21.80 %	269	78.20 %	

* = *p* < 0.05;

** = *p* < 0.01;

*** = *p* < 0.001.

1 Test statistics examined include the Wicoxon-Mann-Whitney test statistic calculated for interval or ordinal variables, while Chi-square and Fisher’s exact test are calculated for categorical variables.

2 Marital status is significantly different between participants reporting no HM ties and one or more HM ties only according to the Fisher’s exact test, which is appropriate for small sample sizes; the chi-square test is not significant for marital status.

**Table 3.** Poisson regression predicting health matters network size at follow-up with baseline demographic and health status/behavior measures

Variables	Model 1	Model 2	Model 3	Model 4	Model 5
Demographics					
Age (tens of years)	0.93 (0.04)	0.95 (0.04)	0.92 (0.04)	0.93 (0.04)	0.96 (0.04)
Married/Living as Married	1.41 (0.17) **	1.41 (0.18) **	1.45 (0.18) **	1.47 (0.18) **	1.40 (0.18) **
Income (tens of thousands)	1.00 (0.01)	1.00 (0.01)	1.00 (0.01)	1.00 (0.01)	1.00 (0.01)
Education (years)	1.00 (0.02)	0.99 (0.02)	1.01 (0.02)	1.01 (0.02)	0.98 (0.02)
Criminal Justice Status					
Prison	0.72 (0.09) **	—	—	—	0.77 (0.10) *
Probation	0.85 (0.10)	—	—	—	0.91 (0.11)
Substance Use					
Alcohol Use (Intoxication)	—	1.33 (0.17) *	—	—	1.28 (0.17)
Crack/Cocaine Use	—	0.76 (0.11) *	—	—	0.75 (0.11) *
Daily Tobacco Use	—	0.83 (0.08)	—	—	0.87 (0.09)
Physical Health					
BMI	—	—	1.00 (0.01)	—	1.00 (0.01)
Exercise (days/week)	—	—	1.00 (0.01)	—	0.99 (0.02)
Other Health Status					
Depression (2 weeks +)	—	—	—	0.84 (0.10)	0.83 (0.10)
Overnight Hospital Stay	—	—	—	0.87 (0.15)	0.90 (0.15)
Number of Observations	341	339	340	340	339
LR Chi^2^	21.11 **	25.13 ***	13.98 *	17.37 **	33.10 **
Pseudo R^2^	0.02	0.03	0.01	0.02	0.03

Incidence rate ratios and standard errors are presented.

* = *p* < 0.05;

** = *p* < 0.01;

*** = *p* < 0.001.

**Table 4.** Regression predicting health matters network mean discussion frequency at follow-up with baseline demographic and health status/behavior measures

Variables	Model 1	Model 2	Model 3	Model 4	Model 5
Demographics					
Age (tens of years)	0.10 (0.06)	0.10 (0.06)	0.07 (0.06)	0.08 (0.05)	0.08 (0.06)
Married/Living as Married	0.05 (0.17)	0.07 (0.17)	0.01 (0.17)	−0.02 (0.17)	−0.05 (0.17)
Income (tens of thousands)	−0.01 (0.01) *	−0.01 (0.01) *	−0.01 (0.01) *	−0.01 (0.01) *	−0.01 (0.01)
Education (years)	−0.01 (0.03)	−0.01 (0.03)	−0.01 (0.03)	−0.01 (0.03)	0.01 (0.03)
Criminal Justice Status					
Prison	0.05 (0.16)	—	—	—	0.06 (0.16)
Probation	0.33 (0.16) *	—	—	—	0.34 (0.16) *
Substance Use					
Alcohol Use (Intoxication)	—	0.33 (0.17)	—	—	0.13 (0.17)
Crack/Cocaine Use	—	−0.18 (0.18)	—	—	−0.11 (0.18)
Daily Tobacco Use	—	0.19 (0.13)	—	—	0.14 (0.13)
Physical Health					
BMI	—	—	0.02 (0.01)**	—	0.02 (0.01)*
Exercise (days/week)	—	—	0.03 (0.02)	—	0.03 (0.02)
Other Health Status					
Depression (2 weeks +)	—	—	—	0.40 (0.15)*	0.32 (0.15)*
Overnight Hospital Stay	—	—	—	0.51 (0.24)*	0.50 (0.23)*
Number of Observations	265	264	264	264	264
F	2.28*	1.54	2.70*	3.31**	2.76**
Adjusted R^2^	0.03	0.01	0.04	0.05	0.08

Coefficients and standard errors are presented;

* = *p* < 0.05;

** = *p* < 0.01;

*** = *p* < 0.001.

**Table 5.** Regression predicting health matters mean encouragement to improve health at follow-up with baseline demographic and health status/behavior measures.

Variables	Model 1	Model 2	Model 3	Model 4	Model 5
Demographics					
Age (tens of years)	0.05 (0.05)	0.05 (0.06)	0.05 (0.05)	0.05 (0.05)	0.03 (0.05)
Married/Living as Married	0.14 (0.18)	0.12 (0.18)	0.04 (0.18)	−0.01 (0.18)	0.04 (0.18)
Income (tens of thousands)	−0.02 (0.01) ***	−0.02 (0.01) ***	−0.02 (0.01) ***	−0.02 (0.01) ***	−0.02 (0.01) **
Education (years)	−0.01 (0.03)	−0.03 (0.03)	−0.03 (0.03)	−0.03 (0.03)	0.01 (0.03)
Criminal Justice Status					
Prison	0.48 (0.16) **	—	—	—	0.48 (0.16) **
Probation	0.39 (0.16) *	—	—	—	0.41 (0.16) *
Substance Use					
Alcohol Use (Intoxication)	—	−0.16 (0.17)	—	—	−0.01 (0.17)
Crack/Cocaine Use	—	−0.01 (0.18)	—	—	0.06 (0.17)
Daily Tobacco Use	—	0.15 (0.13)	—	—	0.04 (0.13)
Physical Health					
BMI	—	—	0.01 (0.01)*	—	0.01 (0.01)
Exercise (days/week)	—	—	0.02 (0.02)	—	0.03 (0.02)
Other Health Status					
Depression (2 weeks +)	—	—	—	0.40 (0.15) **	0.40 (0.15) **
Overnight Hospital Stay	—	—	—	0.46 (0.23) *	0.45 (0.23) *
Number of Observations	265	264	264	264	264
F	4.96 ***	3.03 **	3.96 ***	5.38 ***	3.76 ***
Adjusted R^2^	0.08	0.05	0.06	0.09	0.12

Coefficients and standard errors are presented

* = *p* < 0.05;

** = *p* < 0.01;

*** = *p* < 0.001.

**Table 6.** Logistic regression predicting discussant in health matters network at follow-up with baseline demographic and health status/behavior measures.

Variables	Model 1	Model 2	Model 3	Model 4	Model 5
Demographics					
Age (tens of years)	0.73 (0.09) *	0.78 (0.09) *	0.75 (0.09) *	0.76 (0.09) *	0.74 (0.10) *
Married/Living as Married	2.63 (1.30) *	2.63 (1.30) *	2.68 (1.31) *	3.03 (1.52) *	2.95 (1.54) *
Income (tens of thousands)	1.00 (0.01)	0.99 (0.01)	1.00 (0.01)	0.99 (0.01)	0.99 (0.01)
Education (years)	0.91 (0.06)	0.93 (0.06)	0.95 (0.06)	0.95 (0.06)	0.90 (0.06)
Criminal Justice Status					
Prison	0.52 (0.21)	—	—	—	0.57 (0.24)
Probation	0.37 (0.14) *	—	—	—	0.37 (0.15) **
Substance Use					
Alcohol Use (Intoxication)	—	1.78 (0.76)	—	—	1.70 (0.77)
Crack/Cocaine Use	—	0.58 (0.21)	—	—	0.53 (0.19)
Daily Tobacco Use	—	0.75 (0.22)	—	—	0.91 (0.28)
Physical Health					
BMI	—	—	1.01 (0.02)	—	1.00 (0.02)
Exercise (days/week)	—	—	0.95 (0.05)	—	0.92 (0.05)
Other Health Status					
Depression (2 weeks +)	—	—	—	0.66 (0.21)	0.61 (0.20)
Overnight Hospital Stay	—	—	—	0.42 (0.17)*	0.40 (0.17) *
Number of Observations	341	339	340	340	339
LR Chi^2^	18.57 **	16.32 *	12.65 *	18.28 **	31.89 **
Pseudo R^2^	0.05	0.04	0.03	0.05	0.09

Odds ratios and standard errors are presented

* = *p* < 0.05;

** = *p* < 0.01;

*** = *p* < 0.001.

## References

[R1] (Anderson 2014) Anderson Elijah. 2014 The white space. Sociology of Race and Ethnicity 1: 10–21.

[R2] (Aschenbrenner 1975) Aschenbrenner Joyce. 1975 Lifelines: Black Families in Chicago New York: Holt, Reinhar & Winston.

[R3] (Banks 2011) Banks Ralph Richard. 2011 Is Marriage for White People?: How the African American Marriage Decline Affects Everyone New York: Dutton, Penguin Group.

[R4] (Borrell et al. 2013) Borrell Luis N., Catarina I. Kiefe, Diez-Roux Ana V., Williams DR, and Gordon-Larsen Penny. 2013 Racial discrimination, racial/ethnic segregation and health behaviors in the CARDIA study. Ethnicity and Health 18: 227–43. 22913715 10.1080/13557858.2012.713092 PMC3523091

[R5] (Boyd 1998) Boyd Julia A. 1998 Can I Get a Witness? Black Women and Depression New York: Dutton.

[R6] (Carson 2014) Carson E. Ann. 2014 Prisoners in 2013 Washington: U.S. Department of Justice, Bureau of Justice Statistics.

[R7] (CDC 2013) Centers for Disease Control and Prevention (CDC). 2013 CDC Health Disparities and Inequalities Report—United States, 2013. Centers for Disease Control and Prevention Morbidity and Mortality Weekly Report S62: s1–s186.

[R8] (Chandler 2010) Chandler Daphne. 2010 The underutilization of health services in the black community: An examination of causes and effects. Journal of Black Studies 40: 915–31.

[R9] (Chatters et al. 1989) Chatters Linda, Taylor Robert Joseph, and Neighbors Harold W.. 1989 Size of informal helper network mobilized during a serious personal problem among black Americans. Journal of Marriage and Family 51: 667–76.

[R10] (Choo and Ferree 2010) Choo Hae Yeon, and Myra Marx Ferree. 2010 Practicing intersectionality in sociological research: a critical analysis of inclusions, interactions, and institutions in the study of inequalities. Sociological Theory 28: 129–49.

[R11] (Clear et al. 2001) Clear Todd R, Rose Dina R., and Ryder Judith A.. 2001 Incarceration and the community: the problems of removing and returning offenders. Crime & Delinquency 47: 335–51.

[R12] (Dixon 2009) Dixon Patricia. 2009 Marriage among African Americans: what does the research reveal? Journal of African American Studies 13: 29–46.

[R13] (Domínguez and Watkins 2003) Domínguez Silvia, and Celeste Watkins. 2003 Creating networks for survival and mobility: social capital among African-American and Latin-American low-income mothers. Social Problems 50: 111–35.

[R14] (Dubay and Lebrun 2012) Dubay Lisa C., and Lebrun Lydie A.. 2012 Health, behavior, and health care disparities: disentangling the effects of income and race in the United States. International Journal of Health Services 42: 607–25. 23367796 10.2190/HS.42.4.c

[R15] (Ellison 1990) Ellison Christopher G. 1990 Family ties, friendships, and subjective well-being among Black Americans. Journal of Marriage and Family 52: 298–310.

[R16] (Fowler and Hill 2004) Fowler Dawnovise N., and Hill Hope M.. 2004 Social support and spirituality as culturally relevant factors in coping among African American women survivors of partner abuse. Violence against Women 10: 1267–82.

[R17] (Freudenberg 2002) Freudenberg Nicholas. 2002 Adverse effects of U.S. jail and prison policies on the health and well-being of women of color. American Journal of Public Health 92: 1895–99. 12453803 10.2105/ajph.92.12.1895 PMC1447348

[R18] (Gage 2013) Gage Elizabeth A. 2013 Social networks of experientially similar others: formation, activation, and consequences of network ties on the health care experience. Social Science and Medicine 95: 43–51. 22999229 10.1016/j.socscimed.2012.09.001 PMC3762911

[R19] (Golub et al. 2010) Golub Andrew, Eloise Dunlap, and Ellen Benoit. 2010 Drug use and conflict in inner-city African American relationships in the 2000s. Journal of Psychoactive Drugs 42: 327–37. 21053755 10.1080/02791072.2010.10400695 PMC3743426

[R20] (Hays and Mindel 1973) Hays William C., and Mindel Charles H. 1973 Extended kinship relations in Black and White families. Journal of Marriage and Family 35: 51–57.

[R21] (Hendricks and Wilson 2013) Hendricks LaVelle, and Angie Wilson. 2013 The impact of crack cocaine on Black America. National Forum Journal of Counseling and Addiction 2: 1–6.

[R22] (Heron 2013) Heron Melonie. 2013 Deaths: Leading causes for 2010. National Vital Statistics Reports 62: 1–96. 24364902

[R23] (Hochstetler et al. 2010) Hochstetler Andy, Matt DeLisi, and Pratt Travis C.. 2010 Social support and feelings of hostility among released inmates. Crime & Delinquency 56: 588–607.

[R24] (Holmes-Eber and Riger 1990) Paul Holmes-Eber, and Riger Stephanie. 1990 Hospitalization and the composition of mental patients’ social networks. Schizophrenia Bulletin 16: 157–64. 2333476 10.1093/schbul/16.1.157

[R25] (Jackson 2007) Jackson Fleda Mask. 2007 Race, Stress, and Social Support: Addressing the Crisis in Black Infant Mortality, Research Report Washington: Joint Center for Political and Economic Studies.

[R26] (Jackson and Knight 2006) Jackson James S., and Knight Katherine M.. 2006 Race and self-regulatory health behaviors: the role of the stress response and the HPA axis in physical and mental health disparities. In Social Structure Aging and Self-Regulation in the Elderly Edited by Warner Schaie K and Laura L. Carstensen. New York: Springer, pp. 189–239.

[R27] (Kelly et al. 1997) Kelly Shona, Clyde Hertzman, and Mark Daniels. 1997 Searching for the biological pathways between stress and health. Annual Review of Public Health 18: 437–62. 10.1146/annurev.publhealth.18.1.437 9143726

[R28] (Klag et al. 2005) Klag Stefanie, O’Callaghan Frances V., and Creed Peter Alexander. 2005 The use of legal coercion in the treatment of substance abusers: an overview and critical analysis of thirty years of research. Substance Use & Misuse 40: 1777–95. 16419556 10.1080/10826080500260891

[R29] (Langan and Pelissier 2001) Langan Neal P., and Pelissier Bernadette M.M.. 2001 Gender differences among prisoners in drug treatment. Journal of Substance Abuse 13: 291–301. 11693453 10.1016/s0899-3289(01)00083-9

[R30] (Lee et al. 2014) Lee Hedwig, Wildeman Christopher, Wang Emily A., Matusko Niki, and Jackson James S.. 2014 A heavy burden: the cardiovascular health consequences of having a family member incarcerated. American Journal of Public Health 104: 421–27. 24432879 10.2105/AJPH.2013.301504 PMC3953802

[R31] (Lee and Wildeman 2013) Lee Hedwig, and Christopher Wildeman. 2013 Things fall apart: health consequences of mass imprisonment for African American women. Review of Black Political Economy 40: 39–52.

[R32] (Levi et al. 2009) Levi Jeffrey, Segal Laura M., Rebecca St. Laurent, and David Kohn. 2011 How Obesity Threatens America’s Future 2011 Washington: Trust for America’s Health and The Robert Woods Johnson Foundation.

[R33] (Moloney et al. 2009) Moloney Katherine P., van den Bergh Brenda J., and Moller Lars F.. 2009 Women in prison: The central issues of gender characteristics and trauma history. Public Health 123: 426–30. 19493553 10.1016/j.puhe.2009.04.002

[R34] (Mullings 2014) Mullings Leith. 2014 On Our Own Terms: Race, Class, and Gender in the Lives of African-American Women New York: Routledge.

[R35] (Neighbors and Jackson 1984) Neighbors Harold W., and Jackson James J.. 1984 The use of informal and formal help: four patterns of illness behavior in the Black community. American Journal of Community Psychology 12: 629–44. 6524587 10.1007/BF00922616

[R36] (Nishka et al. 2010) Nishka Richard, Bhuiya Farida, and Xu Jianmin. 2010 National hospital ambulatory medical care survey: 2007 emergency department summary. National Health Statistics Reports 26: 1–32. 20726217

[R37] (Perry 2006) Perry Brea. 2006 Understanding social network disruption: the case of youth in foster care. Social Problems 53: 371–91.

[R38] (Perry and Pescosolido 2015) Perry Brea, and Bernice Pescosolido. 2015 Social network activation: the role of health discussion partners in recovery from mental illness. Social Science & Medicine 125: 116–28. 24525260 10.1016/j.socscimed.2013.12.033 PMC4110193

[R39] (Perry and Pescosolido 2010) Perry Brea, and Bernice Pescosolido. 2010 Functional specificity in discussion networks: the influence of general and problem-specific networks on health outcomes. Social Networks 32: 345–57.

[R40] (Pescosolido 1991) Pescosolido Bernice. 1991 Illness careers and network ties: a conceptual model of utilization and compliance. In Advances in Medical Sociology Edited by Albrecht Gary and Levy Judith. Greenwich: JAI, vol. 2.

[R41] (Pescosolido et al. 1998a) Pescosolido Bernice, Gardner Carol Brooks, and Lubell Keri M.. 1998a How people get into mental health services: stories of choice, coercion, and “muddling through” from “first-timers”. Social Science & Medicine 46: 275–86. 9447648 10.1016/s0277-9536(97)00160-3

[R42] (Pescosolido et al. 1998b) Pescosolido Bernice, Wright Eric R., Margarita Alegría, and Mildred Vera. 1998b Social networks and patterns of use among the poor with mental health problems in Puerto Rico. Medical Care 36: 1057–72. 9674623 10.1097/00005650-199807000-00012

[R43] (Poussaint and Alexander 2000) Poussaint Alvin F., and Alexander Amy. 2000 Lay My Burden Down: Suicide and the Mental Health Crisis Among African-Americans Boston: Beacon Press.

[R44] (Rose and Clear 2003) Rose Dina, and Clear Todd. 2003 Incarceration, re-entry, and social capital: social networks in the balance. In Prisoners Once Removed: The Impact of Incarceration and Reentry on Children, Families, and Communities Edited by Travis Jeremy and Waul Michelle. Washington: The Urban Institute.

[R45] (Sarkisian and Gerstel 2004) Sarkisian Natalia, and Gerstel Naomi. 2004 Kin support among blacks and whites: race and family organization. American Sociological Review 69: 812–37.

[R46] (Schnittker and John 2007) Schnittker Jason, and Andrea John. 2007 Enduring stigma: the long-term effects of incarceration on health. Journal of Health and Social Behavior 48: 115–30. 17583269 10.1177/002214650704800202

[R47] (Schulz et al. 2006) Schulz Amy J., Gravlee Clarence C., Williams David R., Israel Barbara A., Mentz Graciela, and Rowe Zachary. 2006 Discrimination, symptoms of depression, and self-rated health among African American women in Detroit: results from a longitudinal analysis. American Journal of Public Health 96: 1265–70. 16735638 10.2105/AJPH.2005.064543 PMC1483853

[R48] (Smedley et al. 2003). Smedley Brian D, Stith Adrienne Y., and Nelson Alan. R. 2003 Unequal Treatment: Confronting Racial and Ethnic Disparities in Healthcare Washington, D.C.: National Academy Press. 25032386

[R49] (Smith and Christakis 2008) Smith Kristen P., and Christakis Nicholas A.. 2008 Social networks and health. Annual Review of Sociology 34: 405–29.

[R50] (Stack 1974) Stack Carol. 1974 All Our Kin: Strategies for Survival in a Black Community New York: Harper and Row.

[R51] (Thoits 2011) Thoits Peggy A. 2011 Mechanisms linking social ties and support to physical and mental health. Journal of Health and Social Behavior 52: 145–61. 21673143 10.1177/0022146510395592

[R52] (Tuchman 2010) Tuchman Ellen. 2010 Women and addiction: the importance of gender issues in substance abuse research. Journal of Addictive Diseases 29: 127–38. 20407972 10.1080/10550881003684582

[R53] (Umberson et al. 2014) Umberson Debra, Williams Kristi, Thomas Patricia A., Liu Hui, and Mieke Beth Thomeer. 2014 Race, gender, and chains of disadvantage: Childhood adversity, social relationships, and health. Journal of Health and Social Behavior 55: 20–38. 24578394 10.1177/0022146514521426 PMC4193500

[R54] (Wildeman and Western 2010) Wildeman Christopher, and Bruce Western. 2010 Incarceration in fragile families. The Future of Children 20: 157–77. 20964136 10.1353/foc.2010.0006

[R55] (Williams 2003) Williams David R. 2003 The health of men: Structured inequalities and opportunities. American Journal of Public Health 93: 724–31. 12721133 10.2105/ajph.93.5.724 PMC1447828

[R56] (Williams and Jackson 2005) Williams David R. and Pamela Braboy Jackson. 2005 Social sources of racial disparities in health. Health Affairs 24: 325–334. 15757915 10.1377/hlthaff.24.2.325

[R57] (Wilper et al. 2009) Wilper Andrew P., Woolhandler Steffie, Boyd J. Wesley, Lasser Karen E., Danny McCormick, Bor David H., and Himmelstein David U.. 2009 The health and health care of US prisoners: Results of a nationwide study. American Journal of Public Health 99: 666–72. 19150898 10.2105/AJPH.2008.144279 PMC2661478

[R58] (Wilson 2012) Wilson William Julius. 2012 The Truly Disadvantaged: The Inner City, the Underclass, and Public Policy, 2nd ed. Illinois: University of Chicago Press.

[R59] (Woods-Giscombe 2010) Woods-Giscombe Cheryl L. 2010 Superwoman schema: African American women’s views on stress, strength, and health. Qualitative Health Research 20: 668–83. 20154298 10.1177/1049732310361892 PMC3072704

